# Supplementary material for: Synthetic Notch-Receptor-Mediated Transmission of a Transient Signal into Permanent Information via CRISPR/Cas9-Based Genome Editing
Source: Cells. 2020 Aug 20;9(9):1929. doi: 10.3390/cells9091929 (PMC7563181; doi:10.3390/cells9091929)
Supplement: Supplementary file 1 [file cells-09-01929-s001.pdf]

**Supplementary Materials:**

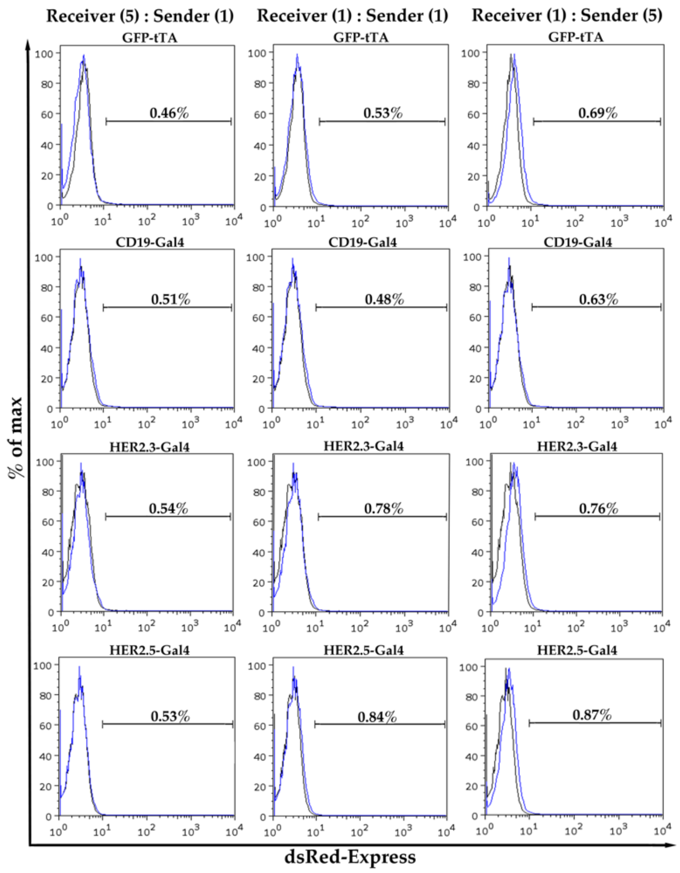


**Figure S1.** Flow cytometry analyses of DsRed-Express fluorescence of all used SynNotch constructs after unspecific activation with HEK293 cells for all investigated ratios.

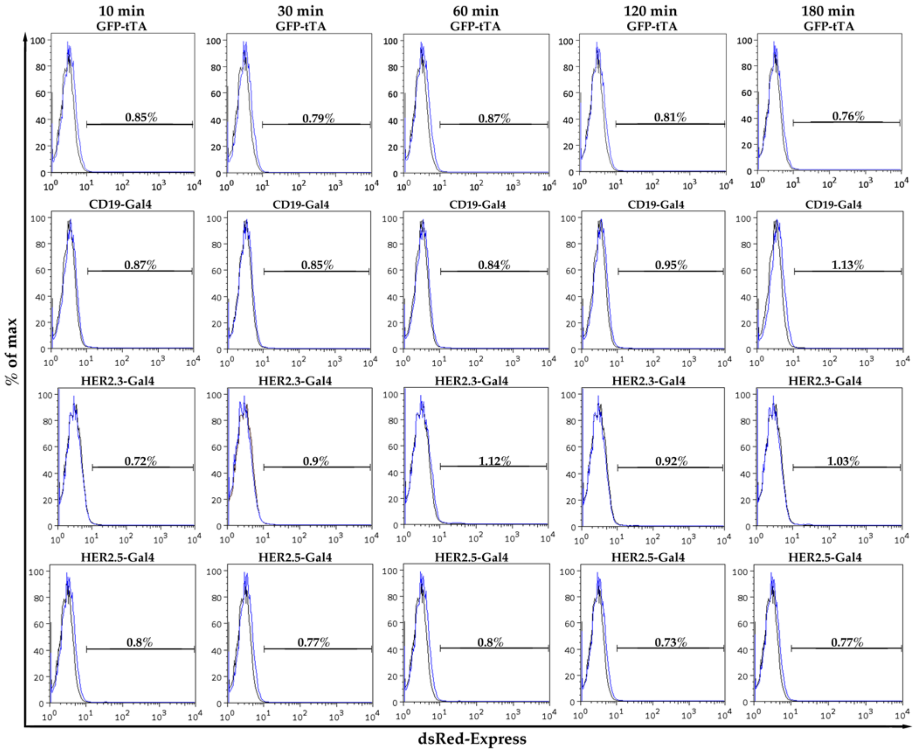


**Figure S2.** Flow cytometry analyses of DsRed-Express fluorescence of all used SynNotch constructs after unspecific activation with HEK293 cells for all investigated time points.

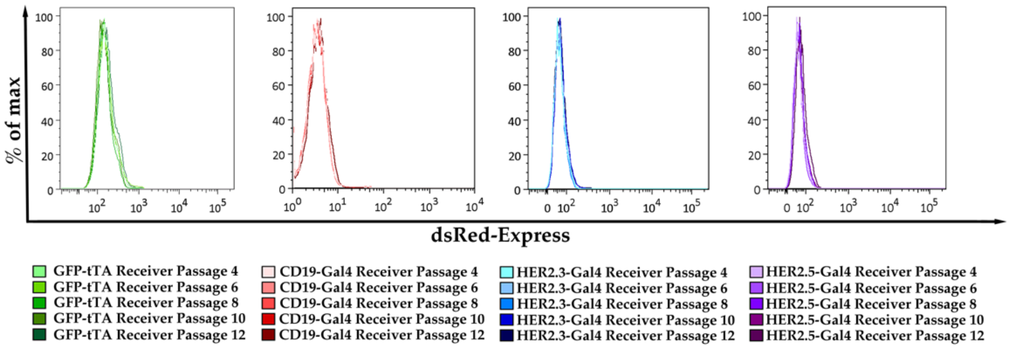


**Figure S3.** Flow cytometry analyses of DsRed-Express fluorescence of all used SynNotch constructs during ongoing culture over 8 passages.
